# Implementation of Targeted Point of Care HIV Testing in a Pediatric Emergency Department

**DOI:** 10.1097/pq9.0000000000000248

**Published:** 2020-01-10

**Authors:** Seema R. Bhatt, Michelle D. Eckerle, Jennifer L. Reed, Venita Robinson, Angela Brown, Joyce Lippe, Carolyn Holland, Srikant Iyer

**Affiliations:** From the * Department of Pediatrics, University of Cincinnati College of Medicine, Cincinnati, Ohio; † Division of Emergency Medicine, Cincinnati Children’s Hospital Medical Center, Cincinnati, Ohio; ‡ Quality Improvement Services, Nationwide Children’s Hospital, Columbus, Ohio; § Kaiser Permanente, Roseville, Calif.; ¶ Division of Emergency Medicine, University of Florida College of Medicine, Gainesville, Fla.; ‖ Department of Pediatrics, Emory University School of Medicine, Atlanta, Ga.; ** Division of Emergency Medicine, Children’s Healthcare of Atlanta, Atlanta, Ga.

## Abstract

HIV infection rates are increasing among adolescents. Despite guidelines recommending annual HIV screening among sexually active adolescents, 3.6% of adolescents tested for other sexually transmitted infections (STI) in a pediatric emergency department (PED) were screened for HIV. The aim was to increase HIV screening to 90%.

**Methods:**

Interventions were designed to address 4 key drivers thought to be critical in reliably offering HIV testing. The primary outcome measure was the proportion of adolescents offered HIV testing among those being tested for common STIs. Statistical process control charts were used to measure performance over time and differentiate common versus special cause variation.

**Results:**

We instituted point of care (POC) HIV testing in the PED in January 2012. The proportion of STI tested patients offered HIV testing was increased to >87% and sustained this performance. Implementation of a clinical decision support tool had the highest impact. The majority offered testing agreed, and the most common reason for refusal was a recent negative test. We identified eleven HIV positive patients over 5 years. Eight were newly diagnosed, and 3 had prior positive tests but were not connected to care. All 11 were successfully connected to providers with HIV care expertise.

**Conclusions:**

POC HIV testing is feasible, acceptable, and sustainable in a PED setting. The implementation of targeted HIV POC testing in the PED increased the number of HIV tests being offered, the number of high-risk patients being screened, and the number diagnosed and connected to care.

## INTRODUCTION

An estimated 1.2 million people live with HIV/AIDS in the United States, and 14% have undiagnosed infections.^1^ Twenty-one percent of new HIV infections occur among 13- to 24-year olds.^1–3^ High-risk behaviors associated with HIV infection start in adolescence, but testing in this population is inadequate.^4,5^ Adolescents account for ~15% of all emergency department (ED) visits in the United States and 4.6% report utilizing the ED for primary care, rating pediatric emergency departments (PEDs) as a high preference location for HIV testing.^6–8^

The Centers for Disease Control and Prevention (CDC) recommends that comprehensive sexually transmitted infection (STI) testing include HIV screening without requiring written consent and that annual screening is performed for those with risk factors.^9^ The American Academy of Pediatrics (AAP) recommends that high-risk youth be tested for HIV annually and that routine STI testing include HIV screening. The AAP also recommends that EDs and urgent cares in high prevalence areas implement routine HIV testing, and use a negative HIV test as an opportunity to counsel adolescents on the reduction of high-risk behaviors.^10^ Because earlier diagnosis and treatment of HIV leads to a better quality of life and decreased morbidity and transmission risk, screening for HIV infection is cost-effective even at infection prevalence rates of <0.1%.^11–13^

Global aims were to increase knowledge and decrease the spread of HIV by facilitating earlier diagnosis and treatment. Review of PED data before this improvement project showed that 3.6% of patients with a discharge diagnosis of cervicitis, pelvic inflammatory disease, urethritis, or exposure to STI received any testing for HIV. The project aim was to increase the percentage of PED patients being tested for common STIs with a documented offer of HIV testing to 90%.

## METHODS

### Context

Cincinnati Children’s Hospital Medical Center is an urban, tertiary care pediatric academic medical center serving an 8-county area in 3 states. At the time of intervention, the PED had over 89,000 visits annually, and adolescents comprised 21.6% of those visits; 46.9% of adolescent patients were African American, 47.5% White, 54.0% with Medicaid, 39.0% privately insured, and 7.0% self-pay. Cincinnati Children’s Hospital Medical Center PED evaluates ~1,200 patients annually that have an STI-related diagnosis.

The study population included patients being tested for other STIs and excluded patients who denied ever being sexually active or were being evaluated for concerns of sexual assault/abuse.

### Planning the Intervention

The initial project planning was undertaken by a multidisciplinary team composed of physicians, a certified nurse practitioner, nurses, and a project manager. It began by participation in the institution’s Rapid Cycle Improvement Collaborative program, an established program which provides the framework for teams to accomplish focused improvement work. The team met weekly for 6 months to launch the project, including mentored monthly meetings with the institution’s quality improvement leadership. We conducted a failure mode and effects analysis to uncover opportunities for improvement.

Initially identified process barriers to HIV testing included the need for written consent, blood as the only specimen option, delayed turnaround time for results (>24 hours), the absence of a reliable method to deliver results without placing undue burden on ED providers, and the absence of standardized follow-up for preliminary positive patients.

**Fig. 1.**
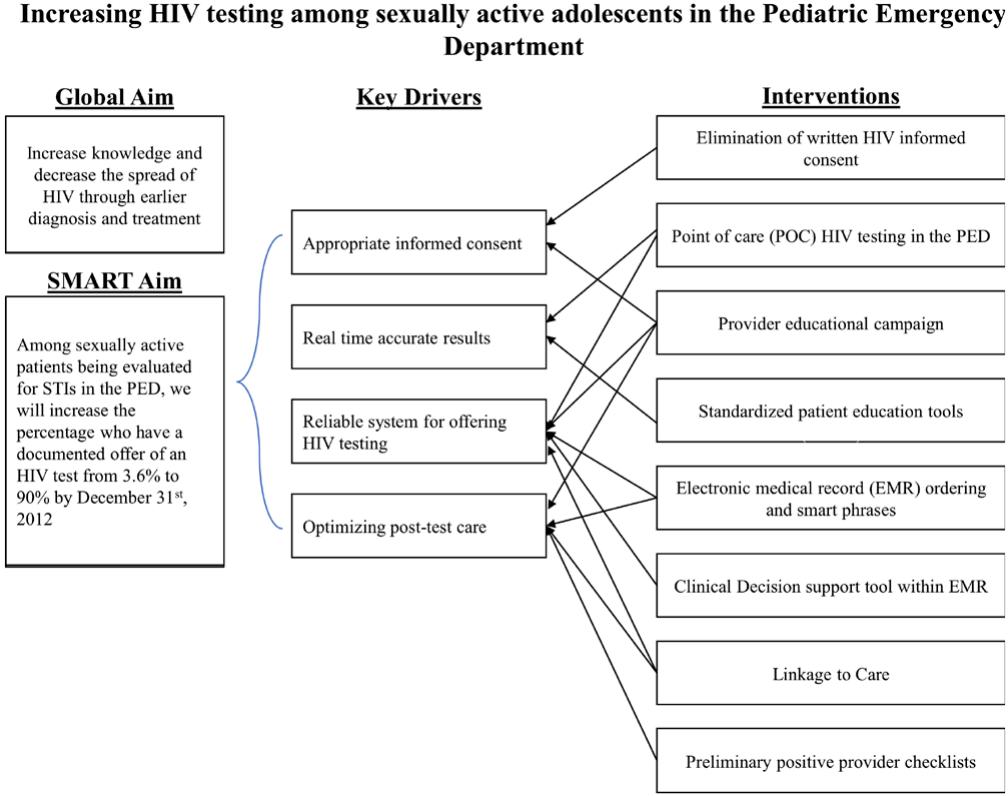
Key driver diagram.

Feedback was solicited from staff and integrated with current existing knowledge of the environment to identify 4 key drivers (Fig. 1) to achieve the aim. Interventions were designed to address these drivers and tested using multiple Plan-Do-Study-Act cycles.

### Key Driver Components

#### Appropriate Informed Consent.

In the 2006 HIV testing recommendations, the CDC endorsed opt-out screening and recommended eliminating the HIV signed consent requirement. Instead, they recommended that general informed consent for medical care encompassed informed consent for HIV testing.^9^ In 2009, state regulations changed to reflect this.

In accordance, collaboration with institutional leadership led to the elimination of the written informed consent requirement. Verbal consent for confidential testing became the new institutional requirement.

#### Accurate Results

Before instituting this program, the test of choice for HIV screening was a fourth generation HIV Antigen/Antibody combination test. This test was performed in the hospital laboratory using a blood sample only. Tests were batched and run once daily. The lack of real-time results was a barrier to appropriate posttest counseling and confirmatory testing contributing to low compliance with testing recommendations.

The quality improvement team requested that the clinical laboratory leadership approve the use of HIV point of care (POC) testing in the PED. Oraquick Advance Rapid HIV 1/2 Antibody Test (OraSure Technologies Inc, Bethlehem, Pa.) was chosen; a Clinical Laboratory Improvement Amendments waived rapid antibody test that provided a result in 20 minutes. This test can be performed on saliva, capillary blood, or whole blood, and test characteristics are comparable between specimens (sensitivity 98.4%–99%, specificity 99.6%–99.9%).^14^

Patient services staff (nurses, paramedics, patient care assistants) already performed POC pregnancy and strep testing. Therefore, the infrastructure already existed to add POC HIV testing efficiently.

We developed a certification program for patient services staff consisting of an online educational module, a written test, and observation of staff running a control test. Sufficient staff were trained to ensure that the test could be run 24 hours a day, 7 days a week. Annual recertification was required, and a PED education specialist was responsible for initial and ongoing certification.

#### Reliable System for Offering HIV Testing

An intensive educational campaign for all care providers, including staff physicians, residents, nurse practitioners, and nurses, was implemented. The focus was to increase awareness of the burden of HIV in the adolescent population as well as the testing recommendations from the CDC and AAP. The importance of incorporating HIV testing into standard STI testing practices was emphasized, and plans to implement this testing were described. Presentations were given at staff and shift change meetings and educational conferences, emails were sent, and we placed flyers/posters throughout the PED.

The need for standardized patient education tools to augment in-person provider counseling was recognized. We purchased an online HIV educational video, which patients viewed while awaiting test results. This video provided information on HIV/AIDS statistics, transmission, symptoms, and safe sex practices. Patients also received pamphlets on HIV and STI facts.

To decrease provider confusion regarding the methods of ordering the HIV test, the *Epic* (2017 Epic Systems Corporation, Verona, Wis.) electronic medical record (EMR) team developed the HIV POC testing order and incorporated it into the existing pre-populated STI order set. Within the order set, POC HIV testing was pre-selected. We also added the POC HIV test order to our EMR preference list.

**Fig. 2.**
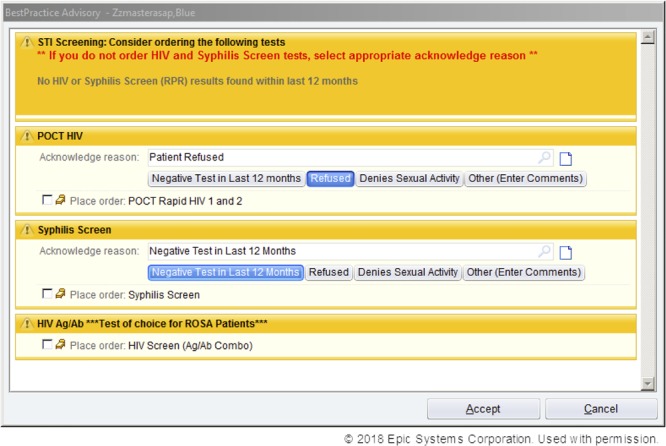
CDS tool screenshot.

Multiple Plan-Do-Study-Act cycles were used to create an EMR POC clinical decision support (CDS) tool (Fig. 2). If a provider ordered any STI testing (eg, gonorrhea, chlamydia, trichomonas, syphilis) and did not order HIV testing, the CDS tool triggered and appeared on the computer screen. This automatic reminder appeared when providers chose not to use the order set or unclicked the order while using the order set. Built into the CDS tool was logic informing the provider of previous HIV testing and the result documented in our EMR within the last 12 months. Providers were asked to choose a reason from a drop-down menu if they did not order HIV testing.

To decrease the burden of documentation, we created smart phases that providers could use to easily document offering HIV testing, results, risk factors, contact information, and counseling.

#### Optimizing Posttest Care

In addition to watching the educational video, all patients with negative test results were given their results in person, provided educational pamphlets, offered condoms, and counseled on re-testing recommendations.

All patients with a preliminary positive result were notified of their result in person stressing that the result was preliminary. In the initial phases of the program, a confirmatory Western blot test was drawn and sent to the Ohio Department of Health.

During the initial implementation period, project team members (HIV team) were responsible for follow-up of preliminary positive patients. The HIV team was notified by paging the on-call HIV team physician or calling a dedicated HIV team phone line and leaving a message. A member of the HIV team was responsible for contacting the patient and arranging for follow-up. If the patient was <18 years of age, the patient would be scheduled to follow-up in 7–10 days at our institutions HIV clinic or their primary care physician’s office to receive confirmatory test results and to establish care. If >18 years of age, follow-up was scheduled with our affiliated adult HIV clinic. The HIV team was responsible for notifying the appropriate follow-up physician and the health department of confirmatory test results. The HIV team was also responsible for contacting the patient and follow-up clinic to ensure that the patient received follow-up care.

After the initial implementation period, we streamlined the follow-up process further. A system was created whereby the hospital laboratory ran confirmatory testing, and the follow-up process was simplified. All preliminary positive patients had confirmatory testing (HIV Antigen/Antibody testing instead of Western blot) sent to the laboratory from the PED. We informed patients that they would be contacted by our HIV Clinic to schedule follow-up regardless of age, at which time they would receive confirmatory test results and further care as indicated. The HIV clinic also took on the responsibility of notifying the health department.

Because preliminary positive tests are a low-frequency event, we created checklists for providers that included reminders to document accurate, confidential contact information in the EMR; to send confirmatory blood testing to the laboratory; to contact the primary care physician, and to notify the HIV team of the preliminary positive result through the EMR messaging system. Additionally, an automated EMR report of all HIV test results was generated each day so that the team could confirm that all steps were performed and linkage to follow-up occurred.

### Methods of Evaluation

The project aim was to design and implement a program for targeted PED HIV screening where POC HIV testing would be offered to all patients being tested for other STIs. We used a time-series study design to measure the effects of the intervention on HIV test offering.

The primary outcome measure was the proportion of the study population with a documented offer of an HIV test. We chose a documented offer rather than the proportion of patients tested because (1) patients could choose to decline testing and (2) if a patient was tested within the last 12 months repeat testing may not be necessary. The target was to increase the offer of HIV testing to 90% among patients being tested for other STIs by December 2012. Because documentation of test offer was not available at baseline, 3.6% baseline HIV testing rate was used as a surrogate for test offering.

Secondary outcomes included the proportion of patients eligible for HIV testing that were offered a test or were tested in the past 12 months regardless of whether it was documented, the HIV positivity rate (incidence) in our population, and the number of preliminary positive patients linked to follow-up care.

Balancing measures included PED length of stay (LOS) and false-positive rate. The goal was to seamlessly incorporate testing into ongoing PED clinical care without adversely affecting PED flow or unnecessarily harming the patient.

### Analysis

Data for all measures were abstracted from the EMR. The proportion of patients offered HIV testing, and the proportion of patients having testing performed were initially plotted as weekly data points separate over and time on a p chart, a specific type of statistical process control chart. We evaluated the incidence of HIV-positive tests in the PED as a proportion of confirmed positive tests among the total number of tests performed. The balancing measure of PED LOS was plotted as a weekly average LOS on an I chart. We used standard rules for the interpretation of statistical process control charts to identify common and special cause variation.

### Human Subject Protection

The Institutional Review Board determined that this was a quality/process improvement project, solely for the benefit of participants, and not human subjects’ research. Therefore, the project did not require Institutional Review Board oversight.

## RESULTS

**Fig. 3.**
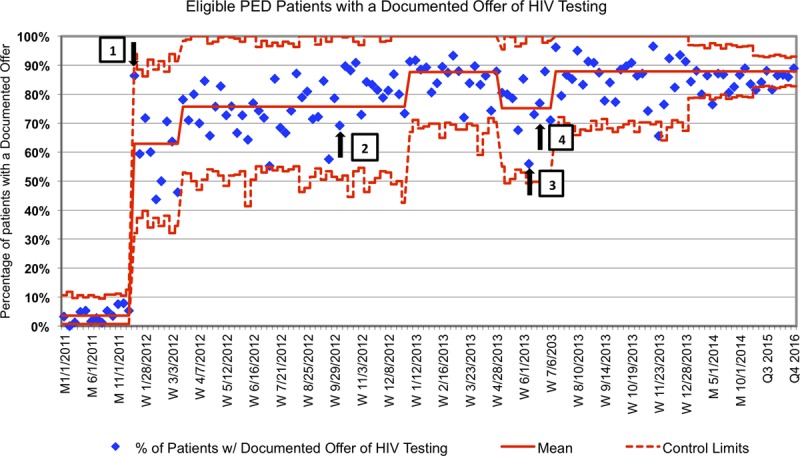
Percentage of eligible PED patients with a documented offer of HIV testing. Weekly percentages for 2012–2013, monthly for 2014, and quarterly for 2015–2016.

At baseline, HIV testing was a rare event. We implemented POC HIV testing in January 2012, and within 2 months, the percent of targeted patients who had a documented offer of an HIV test increased to 75%. Rates then appeared to stabilize despite multiple educational interventions. A higher reliability intervention was needed to achieve the goal. We created the CDS tool, and it went live in October 2012. Comparing the 6-month period before and after implementation of the CDS, documented offers of testing rose from 75% to 87% and were sustained until June 2013 when operational changes to the flow of the PED (STI testing no longer performed at Urgent Care sites where scribes were present to remind providers to order testing) resulted in a transient decline. By July 2013, documented offers again increased to 87% and were maintained through 2016, at which time we reduced the frequency of data monitoring (Fig. 3). The most common reason for test refusal was a recent negative test.

**Fig. 4.**
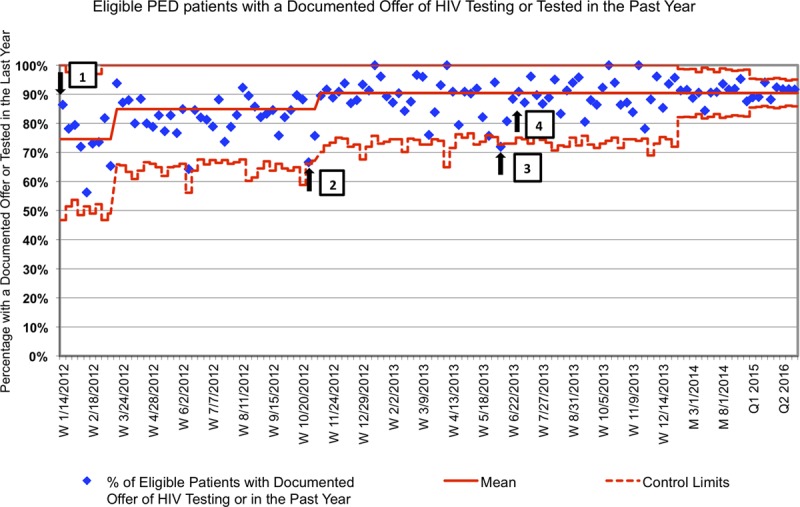
Percentage of eligible PED patients with a documented offer of HIV testing or tested in the past year. Weekly percentages for 2012–2013, monthly for 2014, and quarterly for 2015–2016.

The proportion of eligible patients offered testing or with a documented result within the last 12 months increased to ~ 85% within 2 months of implementation of POC HIV testing. A similar plateau occurred until the introduction of the CDS tool in October 2012 at which time it increased to 90%, sustained through 2016. (Fig. 4)

From January 2012 to December 2016, we tested 4,378 patients. Eleven positive patients were identified in the PED during that time, resulting in a positivity rate of 0.25%. Three of those patients had prior positive tests but were not receiving care and did not disclose their status to PED providers before testing. Through our intervention, 100% of patients were linked to follow-up care.

We collected LOS data for the target population from January 2012 to November 2012. LOS was not affected. The median LOS for those tested for HIV was 1.7 minutes less than those that did not get HIV testing but did receive other STI testing.

There were 3 false-positive test results in addition to the 11 true positives, equating to a false positive rate of 0.069%.

## DISCUSSION

Using the Model for Improvement, we dramatically increased the rate of HIV testing among the target population according to national recommendations. Despite perceived barriers before implementation, we demonstrated that testing could be integrated into routine delivery of care in a relatively short period. HIV screening was systematically embedded into routine STI screening procedures in the PED, and changes were sustained.

Prior studies of HIV screening programs in a PED setting have reported modest successes with differing approaches. These programs still required written consent, testing was performed only on blood samples, testing was offered at limited times, and immediate results were not available. Instead, additional follow-up was required to receive results.^15,16^

Implementing a CDS within the EMR proved to be the most impactful intervention in nearing the goal of 90% of eligible patients being offered HIV testing, likely because the testing order was embedded within the prompt rather than containing a reminder only. The tool assists with accurate identification by providing real-time prompting to the provider to consider HIV testing when they order other STI testing. It also provides a prompt to ensure the correct HIV test is ordered. Minniear et al^17^ showed increases in their testing rates when they introduced a computerized prompt as well.

More recently published studies have shown provider knowledge of testing recommendations is poor, and there are still many perceived barriers to testing.^18,19^ Adolescents continue to have a low perception of risk and, therefore, will often opt out of testing.^20,21^ Many missed opportunities for earlier HIV diagnosis exist as adolescents are still routinely being diagnosed with later-stage disease.^22,23^

Likely, several factors contribute to our inability to achieve the goal of 90%. These include having a large pool of rotating trainees who may not have received education regarding this program. Additionally, the CDS tool is not a hard stop. It can be exited without documenting or ordering testing. A documented offer of testing was the primary outcome. Alternatively, measuring those that received testing, had a documented reason for refusal, or had testing done in the past 12 months, we achieved the goal.

There are limitations to the study. It was implemented at a single center and may not be generalizable to other populations, particularly if there are significant demographic or epidemiologic differences. Also, the accuracy of the data relies on individual provider documentation. The outcome of interest was the appropriate documented offer of an HIV test to an eligible patient. It is possible that a provider offered a test and did not document the offer or used the order set inaccurately, thus documenting an offer when it was not discussed.

## CONCLUSIONS

Key interventions critical to achieving the primary outcome of increasing HIV testing offers according to CDC and AAP recommendations were identified, designed, and implemented. Based on these results, targeted HIV screening can be efficiently and effectively implemented in a PED setting and is beneficial even in low prevalence areas. Further studies should focus on universally offered HIV screening in the adolescent and young adult population in the PED setting.

## ACKNOWLEDGMENTS

The implementation of POC HIV testing (January 2012–December 2017) was supported by a grant from the Ohio Department of Health (HIV testing in Ohio Emergency Departments Grant# 03130012HT0314)

## Disclosure

The authors have no financial interest to declare in relation to the content of this article.
